# Case Report: Penetrating Thoracic Trauma by A Gunshot Involving the Heart

**DOI:** 10.12688/f1000research.54428.1

**Published:** 2021-07-05

**Authors:** Alok Atreya, Ritesh G. Menezes, Ashal Timalsina, Geeta Bashyal, Lokaratna Gyawali, Sushila Gyawali

**Affiliations:** 1 Department of Forensic Medicine, Lumbini Medical College and Teaching Hospital, Tansen, Palpa, Lumbini, 32500, Nepal; 2 Forensic Medicine Division, Department of Pathology, College of Medicine, Imam Abdulrahman Bin Faisal University, Dammam, Saudi Arabia; 3 Lumbini Medical College and Teaching Hospital, Tansen, Palpa, Lumbini, 32500, Nepal; 4 District Hospital, Tansen, Palpa, Lumbini, 32500, Nepal

**Keywords:** entry wound, exit wound, Forensic Pathology, gunshot, hemothorax, Nepal, smooth bore

## Abstract

Firearm related mortality is not frequently encountered in a country like Nepal where there us a stringent law prohibiting buying, selling, carrying or storing of firearms. It is required to have a valid license to have a firearm in possession. Wounds produced by firearm have a typical characteristic the knowledge of which helps to identify the type of firearm used, range of fire, position of the victim and whether the manner of death was homicidal, suicidal or accidental. The present case is a first autopsy-based study from Nepal which discusses the wounds produced by firearm with interpretation of such findings for medicolegal purposes. The present case highlights a social problem where the victim, a psychiatric patient, had no access to prescription medication due to coronavirus disease (COVID-19) related lockdown.

## Introduction

There are stringent laws regarding firearms in Nepal. However, illegal possession of firearms is not uncommon in rural part of this mountainous country. Self-suspension by hanging and consumption of agricultural poisons are the common methods of suicides in Nepal. Use of firearms for committing suicide is rare. We report a case of firearm related fatality from Nepal, where a flint-lock type muzzle loader smooth bore firearm was used to commit suicide and discuss the interpretation of firearm related wounds during a medicolegal examination. The victim in the present case was a schizophrenic patient, who had to discontinue his prescription medication due to coronavirus (Covid-19) related lockdown.

## Case report

**Figure 1.**
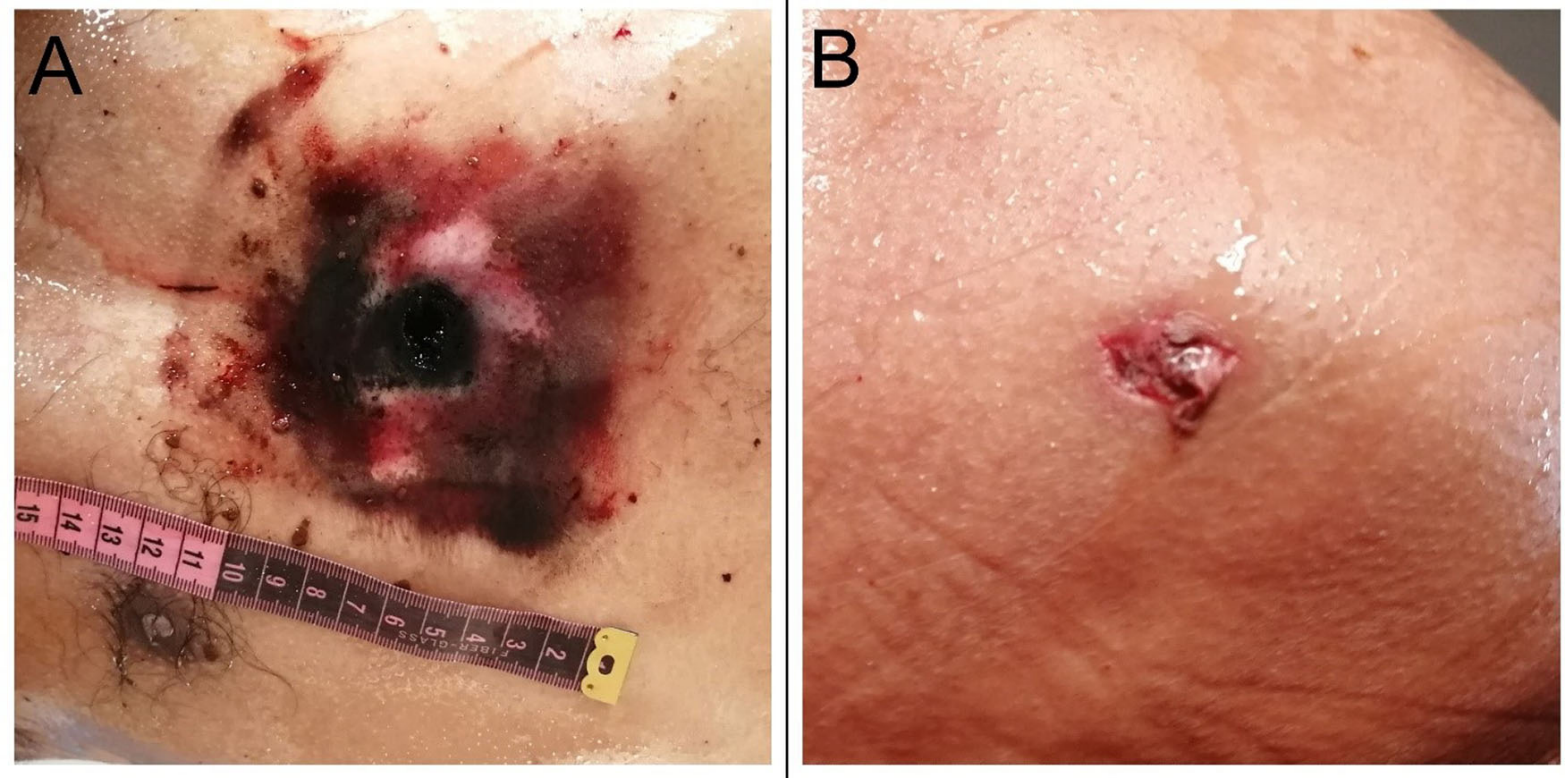
A, An oval entry wound is present on the front of the chest. B, A linear, slit-like lacerated exit wound on the back.

The dead body of 45-year-old male was brought for autopsy. The body was stiff at all the joints. The hands were clenched and the whole body smeared in blood. Post mortem lividity could not be appreciated. A rectangular contusion was noted in the front of the chest which measured 10 × 8 cm. An oval perforated lacerated wound (entry wound) having 1.8 cm diameter was present in the middle of the contusion [ Figure 1]. The wound was charred, black in color with surrounding black tattooing. The perforated wound was 48 cm from the vertex in midline, 115 cm from the sole of the foot and 18 cm from the supra sternal notch. It was 17 cm from the right mid axillary line and 20 cm from the left mid axillary line. A linear slit-like laceration (exit wound) was present on the back in the left side which measured 0.5 × 0.2 cm and was located 25 cm from the vertex, 129 cm from the sole of the foot [ Figure 1].

**Figure 2.**
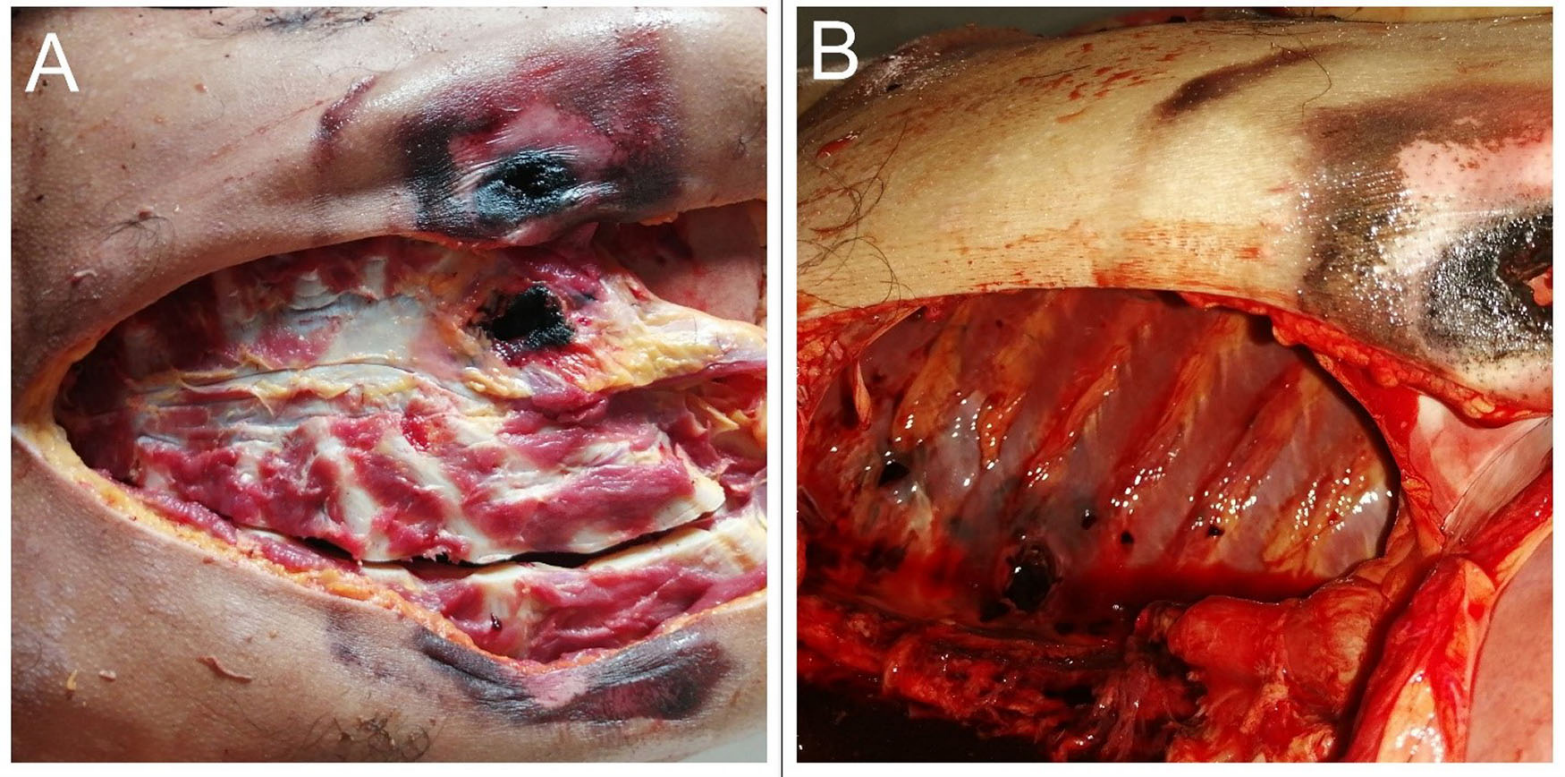
A, A perforating fracture in the xiphoid process. B, Punctured laceration is seen in the posterior chest wall.

**Figure 3.**
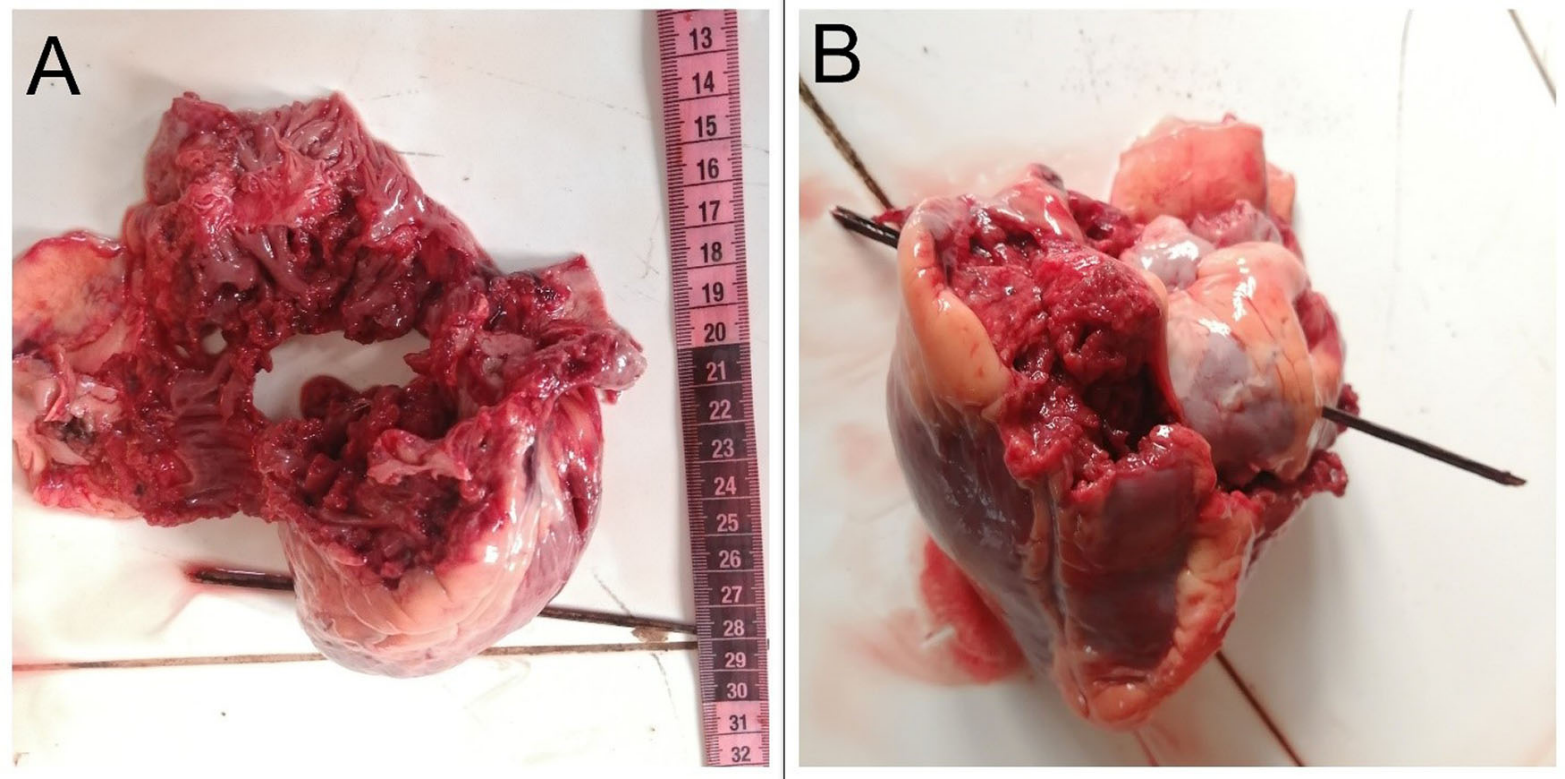
A, Pulverized heart. B, A wooden probe passed through the defect showing the direction of the bullet.

On opening the chest, a perforating fracture was noted in the xiphoid process [ Figure 2]. The heart was pulverized [ Figure 3] with exsanguination of the blood into pericardial cavity. The clotted blood in the pericardial cavity weighed about 650 gm. When a probe was directed through the heart from the defect, it was noted that the entry point was located on the right side of the anterior interventricular septum and exited through the left ventricle [ Figure 3]. The direction was right to left and below upwards. The left lung was completely collapsed and there was a penetrating injury noted at the upper lobe. The rest of the findings were unremarkable.

As per the history provided by the victim’s brother, the deceased was an unmarried male. He was diagnosed with schizophrenia since adolescence for which he was on the prescription medicine Quetiapine. The medicine was not available at his rural pharmacy and due to the Covid-19 pandemic lockdown, he was unable to visit the tertiary hospital for his regular prescription. The medicine was therefore discontinued for 3 months prior to death. The victim consumed homemade arrack (a distilled alcoholic drink) regularly which they prepared at their home.

**Figure 4.**
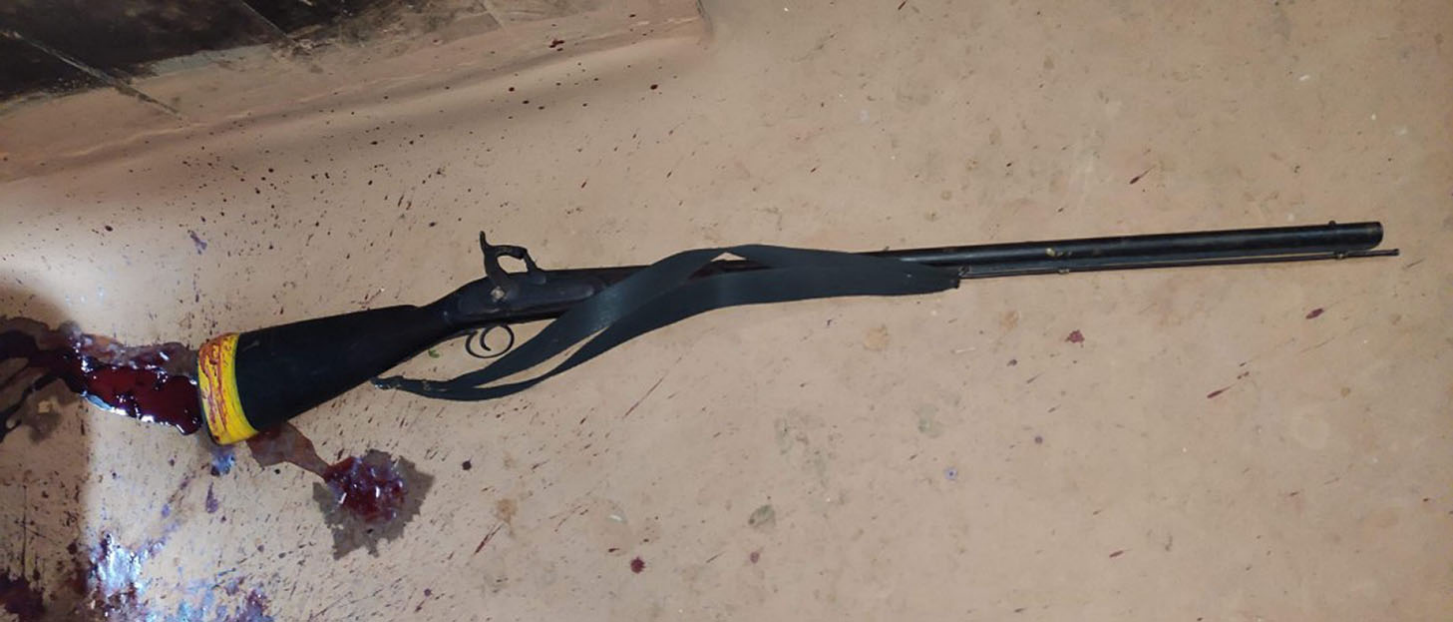
The firearm recovered from the scene of crime.

The crime scene photograph provided by the investigating officer showed a single barrel, smooth-bored firearm which was a flint lock type muzzle loader [ Figure 4].

## Discussion

Shotgun wounds vary in characteristics depending upon type, distance, position and the number of shots. On entering the body cavity, pellets follow the tissue of least resistance. ^1^ A shotgun wound in the chest cannot point well towards the manner of death. However, contact shots are more likely to be suicidal than homicidal and are more fatal for single shot. ^2^

Shots to the heart are very common, with only the head being a more common target. ^3^ Suicidal victims are aware of the lethality of gunshots to this vital organ and some victims even locate the cardiac impulse before taking the shot. Furthermore, suicidal fire is more likely to be directed right to left with homicidal direction commonly being left to right. ^4^

The crime scene visit in cases of firearm related mortality is equally important in interpreting the manner of death. In suicides, the offending weapon is usually found at the scene of crime in contrast to homicides where the perpetrator usually carries away the weapon after committing the crime.

In contrast to stab wounds, gunshot cause more exsanguination of blood due to jagged tearing of myocardium. 60 ml to 200 ml of clotted blood is enough to cause death. Penetrating thoracic trauma as a result of gunshot injuries are associated with traumatic hemothorax, hemopneumothorax, or pneumothorax. ^5^ If the penetrating injury involves the heart, the chances of survival are less than 1%, ^5^ as seen in our case. There are increased number of suicides from Nepal reported during the COVID-19 pandemic. ^6^ The patients with underlying psychiatric disorder could not get access to their prescription medication due to the lockdown as all the public transportation was halted and people were afraid to get out of home. Discontinuation of medication in schizophrenic patients has shown to exacerbate the syndrome. ^7^ This might have been the underlying reason for suicide in the present case. The present case further highlights the social concern the pandemic has brought where patients are restricted of access to their health care needs due to lack of transport in the lockdown.

## Conclusion

Firearm fatalities are medicolegal cases where the autopsy surgeon is required to determine the manner of death based upon the injuries present over the body. Meticulous examination of the wound not only gives clues about the entry and exit wounds but also the range of fire and the weapon used. The manner of death can also be interpretated from the position and characteristics of the wound. A crime scene visit is also mandated in firearm related fatalities which will further help to corroborate the findings.

## Authors’ declaration statements

The authors’ guarantee that the work is original and does not infringe copyright or other party’s property rights. All authors have read and approved this submission and have given appropriate credit to everyone who participated in this work.

## Ethics approval and consent to participate

### Patient consent

Written informed consent was obtained from the deceased patient’s elder brother for publication of this case report.

## Data availability

All data underlying the results are available as part of the article and no additional source data are required.

## Authors’ contributions

AA conceptualized the study and wrote the first draft. RGM and AT reviewed the literature and revised the manuscript. GB, LG and SG conducted the autopsy, provided the case information, and revised the manuscript. All authors read and approved the final version of the manuscript.

## References

[1] Ordog GJ Wasserberger J Balasubramaniam S: Shotgun wound ballistics. *J Trauma.* 1988;28(5):624–631. 10.1097/00005373-198805000-00011 3285016

[2] Cave R DiMaio VJ Molina DK: Homicide or suicide? Gunshot wound interpretation: a Bayesian approach. *Am J Forensic Med Pathol.* 2014;35(2):118–123. 10.1097/PAF.0000000000000085 24781397

[3] Avis SP: Suicidal gunshot wounds. *Forensic Sci Int.* 1994;67(1):41–47. 10.1016/0379-0738(94)90410-3 8082859

[4] Strajina V Živković V Nikolić S: Forensic issues in suicidal single gunshot injuries to the chest: an autopsy study. *Am J Forensic Med Pathol.* 2012;33(4):373–376. 10.1097/PAF.0b013e31824a4797 22354080

[5] Zeiler J Idell S Norwood S: Hemothorax: A Review of the Literature. *Clin Pulm Med.* 2020;27(1):1–12. 10.1097/CPM.0000000000000343 33437141 PMC7799890

[6] Pokhrel S Sedhai YR Atreya A: An increase in suicides amidst the coronavirus disease 2019 pandemic in Nepal. *Med Sci Law.* 2021;61(2):161–162. 33036544 10.1177/0025802420966501

[7] Liu-Seifert H Adams DH Kinon BJ: Discontinuation of treatment of schizophrenic patients is driven by poor symptom response: a pooled post-hoc analysis of four atypical antipsychotic drugs. *BMC Med.* 2005;3:21. 16375765 10.1186/1741-7015-3-21 PMC1327673

