# Supplementary material for: Case Report: Penetrating Thoracic Trauma by A Gunshot Involving the Heart
Source: F1000Res. 2021 Aug 26;10:532. Originally published 2021 Jul 5. [Version 2] doi: 10.12688/f1000research.54428.2 (PMC8420887; doi:10.12688/f1000research.54428.2)
Supplement: Peer review history

# Peer review: Reviewer response for version 2

**DOI:** 10.5256/f1000research.59276.r92855

**Published:** 2021-09-02

**Authors:** Navneet Ateriya

**Affiliations:** 1 Department of Forensic Medicine and Toxicology, All India Institute of Medical Sciences, Gorakhpur, Gorakhpur, Uttar Pradesh, India

No further comments.

Are enough details provided of any physical examination and diagnostic tests, treatment given and outcomes?

Yes

Is the case presented with sufficient detail to be useful for other practitioners?

Yes

Is sufficient discussion included of the importance of the findings and their relevance to future understanding of disease processes, diagnosis or treatment?

Yes

Is the background of the case’s history and progression described in sufficient detail?

Yes

Reviewer Expertise:

Forensic Pathology, Forensic Ballistics

I confirm that I have read this submission and believe that I have an appropriate level of expertise to confirm that it is of an acceptable scientific standard.

# Peer review: Reviewer response for version 2

**DOI:** 10.5256/f1000research.59276.r92854

**Published:** 2021-08-31

**Authors:** Panagiotis K. Stefanopoulos

**Affiliations:** 1 The First Department of Surgery, 401 Army Hospital, Athens, Greece; 2 Military College of Healthcare Administration, Greek Army, Athens, Greece

The authors have successfully and meticulously addressed all comments made by the reviewers. They have also convincingly explained the reasons for not encompassing certain suggestions by giving some additional details about the case. Therefore, I recommend that their article is indexed in its present form.

Are enough details provided of any physical examination and diagnostic tests, treatment given and outcomes?

Yes

Is the case presented with sufficient detail to be useful for other practitioners?

Yes

Is sufficient discussion included of the importance of the findings and their relevance to future understanding of disease processes, diagnosis or treatment?

Partly

Is the background of the case’s history and progression described in sufficient detail?

Yes

Reviewer Expertise:

Maxillofacial surgery, ballistic trauma, chemical warfare

I confirm that I have read this submission and believe that I have an appropriate level of expertise to confirm that it is of an acceptable scientific standard.

# Peer review: Reviewer response for version 1

**DOI:** 10.5256/f1000research.57912.r89003

**Published:** 2021-08-02

**Authors:** Navneet Ateriya

**Affiliations:** 1 Department of Forensic Medicine and Toxicology, All India Institute of Medical Sciences, Gorakhpur, Gorakhpur, Uttar Pradesh, India

The work is presented clearly and accurately by the authors. Flow is good, easy to read and understandable to the readers. There is one one minor correction in Abstract line 1: replace word "us" with "is". Overall, it flows good.

 Introduction is sufficient to give background image of article.

 Case report - Other findings present on skin surrounding fire arm entry wounds such as blackening, tattooing may also be mentioned if present. Author did not mention whether the deceased was subjected to radiological examination prior to autopsy. Images are very helpful in understanding and corelating the findings.

 Discussion part is okay and the conclusions drawn adequately support the findings.

Are enough details provided of any physical examination and diagnostic tests, treatment given and outcomes?

Yes

Is the case presented with sufficient detail to be useful for other practitioners?

Yes

Is sufficient discussion included of the importance of the findings and their relevance to future understanding of disease processes, diagnosis or treatment?

Yes

Is the background of the case’s history and progression described in sufficient detail?

Yes

Reviewer Expertise:

Forensic Pathology, Forensic Ballistics

I confirm that I have read this submission and believe that I have an appropriate level of expertise to confirm that it is of an acceptable scientific standard, however I have significant reservations, as outlined above.

# Response

**Published:** 2021-08-04

**Authors:** Geeta Bashyal

**Affiliations:** Palpa Hospital, Nepal

Dear Reviewer,

 We would like to thank you for your valuable comments. We have corrected the manuscript as instructed. Regarding your query about the radiological examination, due to limited resources at the mortuary radiological examination was not done in the present case. This statement has been added to the manuscript text.

# Peer review: Reviewer response for version 1

**DOI:** 10.5256/f1000research.57912.r89057

**Published:** 2021-07-13

**Authors:** Panagiotis K. Stefanopoulos

**Affiliations:** 1 The First Department of Surgery, 401 Army Hospital, Athens, Greece; 2 Military College of Healthcare Administration, Greek Army, Athens, Greece

The article is very well presented with respect to the case reported, with clear photos that help the reader understand the nature of the lethal injury described therein.

 However, the authors use in their references an article on shotgun wound ballistics, whereas the wound from the flintlock rifle was most likely produced by a single projectile, such as those normally used by this type of weapon (otherwise the authors should clarify whether there were multiple projectiles such as pellets). On the other hand, the literature on wound ballistics as applied to flintlock rifles is virtually non-existent, which is another reason why this article is interesting. 

 The entrance wound, in addition to the description given, appears to show some muzzle imprint, which can be expected from a contact shot, since according to DiMaio's Gunshot Wounds (3rd ed., CRC Press 2016, p. 115) the expanding gases that enter the chest (or the abdominal) cavity cause a bulging out of the skin against the muzzle, resulting in an imprint often significantly larger than the actual diameter of the muzzle. 

 While in hard contact wounds most of the soot is directed into the wound, with little soil of the surrounding skin, in this case the use of black powder in the muzzle loader may account for the extensive blackening of the skin. However, the term "tattooing" used by the authors may be inappropriate, since it is used to indicate the marking of the skin from powder particles, not the blackening from the smoke. 

 An important finding of the autopsy is the complete destruction of the heart. The authors described it simply as pulverized. In my opinion, this is the impressive result of the hydrodynamic pressure generated by the passage of the projectile through the heart, which caused blowout of its walls. I suggest that this is added to their report. 

 Thank you for giving me the opportunity to review this interesting article.

Are enough details provided of any physical examination and diagnostic tests, treatment given and outcomes?

Yes

Is the case presented with sufficient detail to be useful for other practitioners?

Yes

Is sufficient discussion included of the importance of the findings and their relevance to future understanding of disease processes, diagnosis or treatment?

Partly

Is the background of the case’s history and progression described in sufficient detail?

Yes

Reviewer Expertise:

Maxillofacial surgery, ballistic trauma

I confirm that I have read this submission and believe that I have an appropriate level of expertise to confirm that it is of an acceptable scientific standard, however I have significant reservations, as outlined above.

## References

[1] : Gunshot Wounds: Practical Aspects of Firearms, Ballistics, and Forensic Techniques. *CRC Press*.2016;115

# Response

**Published:** 2021-08-04

**Authors:** Geeta Bashyal

**Affiliations:** Palpa Hospital, Nepal

Dear Reviewer,

 We would like to thank you for the analysis of this manuscript and your valuable remarks. The manuscript has been corrected according to the comments. All changes you suggested are incorporated as follows:

**Comment:** However, the authors use in their references an article on shotgun wound ballistics, whereas the wound from the flintlock rifle was most likely produced by a single projectile, such as those normally used by this type of weapon (otherwise the authors should clarify whether there were multiple projectiles such as pellets).

**Response: **The following sentence is added to the text "In the present case as the projectile had exited from the body it could not be ascertained on the type and number of projectiles (pellets)."

**Comment:** The entrance wound, in addition to the description given, appears to show some muzzle imprint, which can be expected from a contact shot, since according to DiMaio's Gunshot Wounds (3rd ed., CRC Press 2016, p. 115) the expanding gases that enter the chest (or the abdominal) cavity cause a bulging out of the skin against the muzzle, resulting in an imprint often significantly larger than the actual diameter of the muzzle. 

**Response: **As per the reviewer's suggestion we did go through the reference and have detailed the entry wound "The entry wound showed some muzzle imprint, signifying a contact shot. The expanding gases that enter the chest cavity cause a bulging out of the skin against the muzzle, resulting in an imprint often significantly larger than the actual diameter of the muzzle. ^3^"

**Comment: **While in hard contact wounds most of the soot is directed into the wound, with little soil of the surrounding skin, in this case the use of black powder in the muzzle loader may account for the extensive blackening of the skin. However, the term "tattooing" used by the authors may be inappropriate, since it is used to indicate the marking of the skin from powder particles, not the blackening from the smoke. 

**Response:** We have corrected the manuscript and have deleted the term tattooing.

**Comment: **An important finding of the autopsy is the complete destruction of the heart. The authors described it simply as pulverized. In my opinion, this is the impressive result of the hydrodynamic pressure generated by the passage of the projectile through the heart, which caused blowout of its walls. I suggest that this is added to their report. 

**Response:** As per the suggestion the following sentence is added to the manuscript "There was complete destruction of the heart noted at autopsy which was due to the hydrodynamic pressure generated by the passage of the projectile through the heart that caused blowout of its walls."
